# Epigenetic Reprogramming for Targeting *IDH*-Mutant Malignant Gliomas

**DOI:** 10.3390/cancers11101616

**Published:** 2019-10-22

**Authors:** Jong-Whi Park, Şevin Turcan

**Affiliations:** Neurology Clinic and National Center for Tumor Diseases, University Hospital Heidelberg, 69120 Heidelberg, Germany; jongwhi.park@med.uni-heidelberg.de

**Keywords:** *IDH*-mutant gliomas, lower-grade gliomas, epigenetic therapies

## Abstract

Targeting the epigenome has been considered a compelling treatment modality for several cancers, including gliomas. Nearly 80% of the lower-grade gliomas and secondary glioblastomas harbor recurrent mutations in isocitrate dehydrogenase (*IDH*). Mutant IDH generates high levels of 2-hydroxyglutarate (2-HG) that inhibit various components of the epigenetic machinery, including histone and DNA demethylases. The encouraging results from current epigenetic therapies in hematological malignancies have reinvigorated the interest in solid tumors and gliomas, both preclinically and clinically. Here, we summarize the recent advancements in epigenetic therapy for lower-grade gliomas and discuss the challenges associated with current treatment options. A particular focus is placed on therapeutic mechanisms underlying favorable outcome with epigenetic-based drugs in basic and translational research of gliomas. This review also highlights emerging bridges to combination treatment with respect to epigenetic drugs. Given that epigenetic therapies, particularly DNA methylation inhibitors, increase tumor immunogenicity and antitumor immune responses, appropriate drug combinations with immune checkpoint inhibitors may lead to improvement of treatment effectiveness of immunotherapy, ultimately leading to tumor cell eradication.

## 1. Introduction 

Gliomas represent the most common primary brain tumors with distinct biological features and clinical behavior, accounting for nearly 80% of the malignant brain tumors [1]. About half of all glioma patients are diagnosed with glioblastoma (GBM) or World Health Organization (WHO) Grade IV glioma, which is one of the most aggressive and untreatable cancers. Current standard of care consists of surgical resection, radiation, and chemotherapy [2]. However, complete resection is virtually impossible because of the highly invasive nature of high-grade gliomas. In addition, extensive inter- and intra-tumor heterogeneity contributes to minimal clinical efficacy.

**Figure 1:**
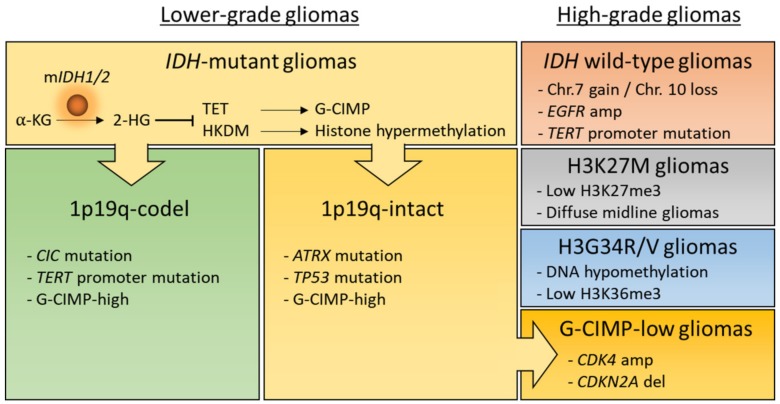
Major subtypes of adult and pediatric gliomas. According to isocitrate dehydrogenase (*IDH*) status, adult diffuse gliomas are largely classified into two subgroups, *IDH*-mutant or wild-type gliomas. Lower grade or *IDH*-mutant gliomas are further classified into two subtypes, 1p19q-codel or -intact gliomas. Importantly, loss of DNA methylation in *IDH*-mutant and 1p19q-intact gliomas during malignant recurrence may involve the progression to high-grade G-CIMP-low gliomas. 2-HG, D-2-hydroxyglutarate; α-KG, α-ketoglutarate; Chr, chromosome; G-CIMP, glioma-CpG island methylator phenotype; H3, Histone 3; HKDM, histone lysine demethylase; m*IDH1/2*, mutant *IDH1/2*; TET, ten-eleven translocation protein.

The classification of gliomas has been mostly based on histological features for many years. However, such a classification limits prediction of clinical outcomes because of the morphological similarities and high inter-observer variability. Recently, extensive molecular profiling provided us with deeper insights into tumor classification and advanced our understanding of gliomas [3,4,5,6,7,8,9]. Consequently, the fourth edition of WHO classification of central nervous system (CNS) tumors integrated phenotypic and genotypic parameters, including the isocitrate dehydrogenase (*IDH*) mutations, the co-deletion of chromosome arms 1p and 19q (1p/19q codel) and histone H3 alterations [10]. According to the molecular signatures, selected gliomas are classified as follows: (1) *IDH*-mutant and 1p/19q codel, oligodendroglioma (WHO grade II-III); (2) *IDH*-mutant, astrocytoma (WHO grade II-III); (3) *IDH*-mutant, glioblastoma (WHO grade IV); (4) *IDH*-wildtype, glioblastoma (WHO grade IV); (5) H3K27M-mutant, diffuse midline glioma (WHO grade IV) (Figure 1). 

Malignant glioma cells exhibit several pathogenic mechanisms involved in tumor growth, maintenance, and drug resistance [11,12]. Alongside known genetic alterations, the initiation and progression of cancers are closely associated with epigenetic abnormalities, including DNA methylation, histone modifications, noncoding RNA (ncRNA), microRNA (miRNA), and chromatin remodeling [13]. In this regard, the initiating genetic alteration such as *IDH* mutations may cooperate with subsequent epigenetic events during malignant progression to a higher grade coupled with a worse prognosis [14,15,16]. Given the dynamic and reversible nature of epigenetic modifications, epigenetic modifiers that regulate these changes have emerged as potential therapeutic targets for the treatment of gliomas. Here, we focus on the dynamic relationships of epigenetic alterations in *IDH*-mutant gliomas. Moreover, glioma patients carrying *IDH* mutations show a favorable prognosis compared to their wild-type counterparts [8,17]. The presence of *IDH* mutations provides a wider window of opportunity for therapeutic interventions.

## 2. Epigenetic Modifications in Gliomas

Epigenetic processes consist of changes in DNA methylation, histone modification, and chromatin states. The physiological form of epigenetic control can be dysregulated in response to pathological signals that are closely linked to gene activity such as activation of oncogenes and silencing of tumor suppressor genes [18]. In almost all cancers, multiple genetic mutations and epigenetic alterations can cooperate with each other. For example, frequent mutations have been found in genes that encode for epigenetic modifiers, potentially leading to altered methylation of cytosine-guanine dinucleotides (CpGs), histone modifications, and ultimately malignant transformation. Conversely, DNA hypomethylation is strongly associated with genomic instability [19,20]. 

### 2.1. IDH-Mutation Induced DNA Hypermethylation

DNA methylation is a common epigenetic modification that occurs in cancers. Increasing evidence suggests that aberrant DNA methylation leads to the occurrence and progression of malignancies [21]. There are three DNA methyltransferases (DNMTs), DNMT1, 3a, and 3b, that produce 5-methylcytosine (5mC) by transferring a methyl group to cytosine. DNMT1 is critical in maintaining DNA methylation [22], whereas DNMT3a and 3b contribute to de novo methylation [23]. DNA methylated sites can be bound and recognized by methyl cytosine binding proteins (MBDs) [24]. Conversely, ten-eleven translocation (TET) proteins are actively involved in DNA demethylation by the oxidation of 5mC to 5-hydroxymethylcytosine (5hmC), 5-formylcytosine (5fC), and 5-carboxylcytosine (5caC) [25]. 

*IDH* encodes isocitrate dehydrogenase that converts isocitrate to α-ketoglutarate (α-KG). More than 80% lower-grade gliomas (LGGs, WHO grade II–III) have *IDH* mutations [5], which are the earliest somatic events in the development of LGGs [15,16,26]. *IDH1/2* mutations have also been found in several types of cancers, covering enchondroma, chondrosarcoma, angioimmunoblastic T cell lymphoma (AITL), intrahepatic cholangiocarcinoma (ICC), and acute myeloid leukemia (AML) [27]. In most *IDH*-mutant cancers, *IDH* mutations are associated with blocked cell differentiation and suppression of immune response [28,29,30]. While less than 5% of CpG sites are hypermethylated in other *IDH*-mutant cancers, approximately 20% of CpG sites are hypermethylated in *IDH*-mutant gliomas compared to their wild-type *IDH* counterparts, suggesting that gliomas with *IDH* mutations have unique molecular features [31]. As noted above, *IDH*-mutant and 1p19q codel gliomas show favorable patient outcomes compared to the wild-type *IDH* or 1p19q-intact gliomas [8].

In gliomas, mutations of the cytosolic *IDH1*, most frequently occurring heterozygous *IDH1* R132H point mutation, are much more common than mutations of the mitochondrial homolog *IDH2* [32]. When *IDH1* or *IDH2* is mutated in gliomas (most frequently at codon R132 or R172, respectively), the mutated protein gains a neomorphic enzymatic activity, and produces D-2-hydroxyglutarate (2-HG) from α-ketoglutarate (α-KG). Consequently, 2-HG inhibits the enzymatic activity of α-KG-dependent dioxygenases, including the TET family of DNA hydroxylases and JumonjiC (JmjC) domain-containing histone lysine demethylases (KDMs), leading to aberrations in numerous biological processes as well as histone and DNA methylation alterations [33,34,35]. Hence, *IDH*-mutant gliomas exhibit genome-wide DNA hypermethylation, resulting in the glioma-CpG island methylator phenotype (G-CIMP) [9]. 

Our findings demonstrate that primary human astrocytes introduced with mutant IDH1 display extensive DNA hypermethylation that recapitulate the patterns observed in G-CIMP-positive LGGs [3]. There is ample evidence that *IDH1* mutation alone is insufficient to initiate glioma formation in vivo [36,37]. In this regard, mutant IDH-driven epigenetic changes make glioma cells more susceptible to additional oncogenic events during multiple clonal evolution [38], as exemplified by 1p19q codel and inactivating alterations in tumor protein p53 (*TP53*), homolog of drosophila capicua (*CIC*) and alpha thalassemia/mental retardation syndrome X-linked (*ATRX*) [39,40].

Despite the slow growth of LGGs, G-CIMP tumors frequently recur with malignant transformation and development of resistance. A recent study has revealed that *IDH*-mutant lower-grade astrocytomas consist of distinct molecular subgroups according to their diverse transcriptomic and methylation data [41]. Additionally, molecular profile analyses demonstrated that loss of methylation in initially G-CIMP-high gliomas is associated with tumor relapse and malignant progression, which explains distinct patterns of epigenetic shifts in longitudinal evolution of lower-grade *IDH*-mutant astrocytic tumors that have intact chromosome 1p/19q [42,43]. Notably, G-CIMP-low subtype with worse clinical outcomes has also been identified in pan-glioma cohorts [4] (Figure 1). 

### 2.2. MGMT Promoter Methylation

In many types of human cancers, the tumor suppressor and DNA repair genes can become transcriptionally inactivated by methylation of cytosine residues in CpG motifs within their promoter regions [44]. The O^6^-methylguanine DNA-methyltransferase (MGMT) enzyme repairs the DNA damage by removing cytotoxic adducts caused by alkylating chemotherapeutic agents. Thus, MGMT promoter methylation is linked to its loss of transcription and reduced DNA repair activity, conferring sensitivity to the cytotoxic effect of alkylating drugs, such as BCNU or temozolomide (TMZ) [45]. Importantly, MGMT promoter hypermethylation has been identified in approximately 40% of gliomas [46]. The prognostic and predictive value of MGMT promoter methylation has been established in GBM [47,48]. Most remarkably, more than 90% of *IDH*-mutant gliomas exhibit hypermethylation of the MGMT promoter [4,49], indicating that these profound epigenetic alterations are strongly associated with *IDH* mutations [50]. Recently, a cogent study demonstrated that the extent of MGMT methylation is a predictive marker for progression-free survival (PFS) of glioma patients harboring *IDH* mutations with TMZ treatment but not radiotherapy [51]. 

### 2.3. Aberrant Histone Modifications in Gliomas

The epigenetic silencing can occur by inappropriate regulation of covalent modifications at one or more amino acids on histones that can potentially alter gene activity. The nucleosome consists of eight histone proteins, two copies each of H2A, H2B, H3 and H4, and 147 base pairs of DNA [52]. Particularly, specific amino acids in the N-terminal tail of each histone is subjected to various histone modifications, including acetylation, methylation, ubiquitination, sumoylation, and phosphorylation. We previously reported that introduction of mutant IDH1 increases the repressive marks such as dimethylated/trimethylated histone H3 lysine 9 (H3K9me2/3) and H3K27me3, as well as active marks such as H3K4me2/3 in immortalized human astrocytes [3,53]. Similarly, in patient samples, increased H3K9me3 staining was observed in *IDH*-mutant and 1p19q codel gliomas [54]. Interestingly, the same study found no association between *IDH* mutation and H3K9me3 staining in astrocytomas or GBM, therefore the differential interplay between histone methylation in 1p/19q codel and 1p/19q non-codel gliomas warrants further investigation. Overall, the significant increase of histone methylation by mutant IDH or 2-HG leads to impaired differentiation [28,37]. 

Histone methyltransferases catalyze methylation reactions at K9 and K27 on histone 3 (H3) by transferring the methyl groups that repress transcription. For instance, methylation of H3K9 is an important epigenetic modification that marks the heterochromatin. While trimethylation (H3K9me3) is involved with Suv39h [55], mono-, and dimethylation (H3K9me1 and H3K9me2) are mostly associated with histone lysine methyltransferase G9a and G9a-like protein (GLP) [56]. BIX-01294 (a diazepin-quinazolin-amine derivative) effectively and specifically inhibits G9a enzymatic activity and lowers H3K9me2 levels in mammalian chromatin [57]. Protein arginine methyltransferase 5 (PRMT5) is one of the PRMT protein family involved in regulation of cell signaling and gene expression [58]. In high-grade gliomas, *PRMT5* is highly expressed, and its expression inversely correlates with patient survival [59]. In vitro and in vivo studies demonstrated that one of PRMT5 inhibitors leads to a non-replicative senescence and apoptosis of glioma cells [60]. 

Importantly, two distinct single-point mutations (K27M and G34R/V) of the *H3* gene variant (*H3F3A*), which encodes the histone variant H3.3, have been reported in pediatric GBM [61]. K27M histone H3.3 mutations are specific to midline gliomas that predominately affect children, while G34R/V histone H3.3 mutations have been exclusively reported in the cerebral hemispheres of mostly adolescent patients [7]. Interestingly, both of *H3F3A* mutations are mutually exclusive with *IDH1* mutations, which are rarely found in pediatric GBM [62,63]. Mechanistically, K27M mutation alters the histone methyltransferase activity of enhancer of zeste homolog 2 (EZH2), the catalytic subunit of Polycomb repressive complex 2 (PRC2), responsible for the methylation of H3K27 [64,65]. Additionally, it has been discovered that EZH2-mediated gene silencing by H3K27me3 is independent of promoter DNA hypermethylation [66]. Given that H3K27me3 is the repressive histone modification, K27M mutation results in reduced levels of H3K27me3 and transcriptional activation [7], thereby leading to global changes in epigenetic state of cells. Accordingly, K27 demethylase inhibitor GSKJ4 as well as inhibitors of histone deacetylases (HDAC) and Bromodomain and extraterminal (BET) proteins have recently shown impressive results in H3K27M-mutant gliomas [67]. 

### 2.4. Chromatin Remodeling

A topologically associating domain (TAD) consists of three-dimensional chromosome regions which are characterized by frequent interactions of DNA sequences within a TAD boundary [68]. TAD boundaries are maintained by the CCCTC-binding factor (CTCF) as an insulator protein that separates TADs from topologically distinct regions by its binding to specific DNA sites [69]. Majority of secondary GBMs harbor *IDH* mutations and platelet-derived growth factor receptor A (*PDGFRA*) alterations [70]. Flavahan et al. reported that DNA hypermethylation disrupts CTCF binding, gene insulation and chromosomal topology, resulting in aberrant expression of oncogenes such as *PDGFRA* [71]. Conversely, in vitro treatment with a DNA demethylating agent reduces DNA hypermethylation at the CTCF motif, which is accompanied by increased CTCF occupancy and restored *PDGFRA* insulation [71].

Loss-of-function mutations of *ATRX*, which encodes the SWI/SNF-like chromatin regulator, occur frequently in *IDH*-mutant adult and pediatric gliomas [8,40,61]. Intriguingly, the epigenetic consequences of Atrx inactivation has been reported in the context of murine neuroepithelial progenitors as a putative glioma cell of origin [72]. Danussi et al. showed that Atrx deficiency leads to altered chromatin accessibility at its vacant binding sites and impaired Atrx-based transcriptional control [72].

## 3. Epigenetic Therapy for Gliomas

In light of the evidence that the disturbance of epigenetic status results in glioma development, the rationale for epigenetic therapy is to reshape the altered methylome toward a non-pathological state [73,74]. Genome-wide molecular-profiling studies, and an improved understanding of epigenome regulation are continuously contributing to spur the development of novel therapeutic strategies for gliomas.

### 3.1. Mutant IDH Inhibitors

Recognition of the critical roles of mutant IDH and subsequent accumulation of 2-HG in glioma development makes mutant IDH an attractive drug target. Accordingly, multiple mutant IDH (both pan- and specific) inhibitors are being tested as targeted therapeutic tools in preclinical and clinical studies of *IDH*-mutant gliomas. Ivosidenib (AG-120, trade name Tibsovo) and Enasidenib (AG-221, trade name Idhifa) have been FDA-approved for use in the treatment of patients with AML, are selective small molecule inhibitors of mutant IDH1 and IDH2, respectively. Notably, potential clinical benefit from ongoing clinical trials of AG-120 and AG-221 has been observed in other hematologic malignancies [75]. In 2016, it has been reported that AG-120 displays a 35% clinical benefit rate and a favorable safety profile in phase 1 trial in *IDH1*-mutant gliomas (ClinicalTrials.gov NCT02073994) [76] (Table 1). In the same year, a trial of AG-221 in *IDH2*-mutant malignancies, including gliomas, was completed, but results have not yet been reported (ClinicalTrials.gov NCT02273739). 

AG-881(Vorasidenib), a pan-inhibitor of both mutant IDH1 and mutant IDH2, exhibits effective brain penetration accompanied by suppression of 2-HG production in an orthotopic glioma model (*IDH1* R132H) [77]. In accordance with this preclinical data, analysis of surgically resected tumors from patients with *IDH1*-mutant gliomas indicate decreased levels of 2-HG following pretreatment with either AG-881 or AG-120, indicating that both inhibitors penetrate into the CNS (ClinicalTrials.gov NCT03343197) [78]. Additionally, AG-881 is in use in a phase 1 trial in *IDH1/2*-mutant gliomas and other solid tumors (ClinicalTrials.gov NCT02481154) [79]. 

BAY1436032, a pan inhibitor of IDH1 R132H, significantly prolongs the survival of human astrocytoma (*IDH1* R132H)-bearing mice [80]. A phase 1 trial is currently ongoing in *IDH1*-mutant advanced solid tumors, including gliomas (ClinicalTrials.gov NCT02746081). Furthermore, other mutant IDH-specific inhibitors such as DS-1001b (ClinicalTrials.gov NCT03030066), IDH305 (ClinicalTrials.gov NCT02381886), and FT-2102(ClinicalTrials.gov NCT03684811) are being utilized in clinical trials on targeted epigenetic agents.

In an early preclinical study of *IDH1*-mutant gliomas, AGI-5198, the first selective mutant IDH1 inhibitor, showed tumor growth inhibition along with astroglial differentiation [81]. However, treatment of preclinical orthotopic models of *IDH1*-mutant gliomas with AGI-5198 is not sufficient to reverse the mutant IDH-driven oncogenic changes such as DNA [81] or histone methylation [82,83]. Additionally, limited efficacy in reducing proliferative capacity is observed upon AGI-5198 exposure in subsequent in vivo [84] and in vitro studies [82,83] for gliomas. These mixed results imply that the role of mutant IDH may be changed from driver to passenger during glioma progression [82]. Moreover, perhaps it is not a surprise that deletion of mutant IDH1 allele can occur during tumor recurrence, resulting in reduced 2-HG levels [85]. In line with this, a recent study showed that CRISPR/Cas9-mediated knockout of IDH1 R132H induces genome-wide DNA demethylation and closely recapitulates G-CIMP-low phenotype in isogenic glioma cell lines [86].

**Figure 2:**
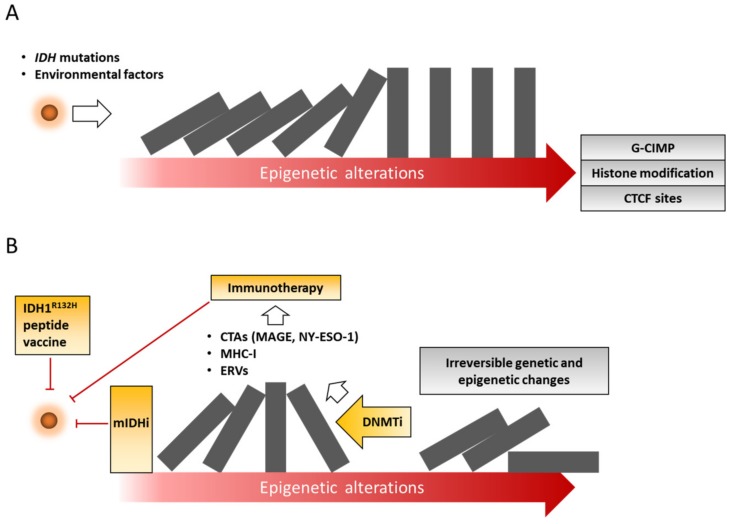
Schematic overview illustrating current approaches to epigenetic therapy for *IDH*-mutant gliomas. (**A**) *IDH* mutations result in epigenetic abnormalities such as DNA methylation, histone modification, and aberrant chromatin states. (**B**) Consequently, mutant IDH-driven epigenetic alterations lead to irreversible genetic and epigenetic changes. Epigenetic drugs and immunotherapy are highlighted in yellow. CTAs, cancer testis antigens; DNMTi, DNA methyltransferases inhibitor; ERVs, endogenous retroviruses; CTCF, CCCTC-binding factor; G-CIMP, glioma-CpG island methylator phenotype; MHC-I, major histocompatibility class-I; mIDHi, mutant IDH inhibitor.

We recently reported that loss of mutant *IDH1* expression is not sufficient to repress tumor growth derived from immortalized human astrocytes expressing doxycycline-inducible mutant IDH1 [53], thus suggesting that *IDH1* mutation is dispensable for glioma progression and maintenance. Given that irreversible genetic and epigenetic alterations occur in mutant IDH-dependent reprogramming during tumor progression [53,85] (Figure 2), determining the timing and the scheduling of the mutant IDH inhibitors may be a critical challenge for maximizing their efficacy in *IDH*-mutant gliomas.

### 3.2. IDH1 Peptide Vaccination

As in the case of mutant IDH inhibitors, a considerable effort has been placed on a therapeutic vaccine against mutant IDH. A proof-of-concept study of IDH1 R132H peptide vaccines has been initiated in a humanized mouse sarcoma model [87]. Importantly, CD4+ T helper cell-mediated antitumor immunity is detected in subcutaneous IDH1 R132H sarcoma-bearing mice immunized with the mutant-specific peptide vaccination [87]. Likewise, Pellegata and colleagues have reported that IDH1 R132H-specific peptide vaccines significantly prolong the survival of mice intracranially implanted with GL-261 glioma cells overexpressing mutant IDH1 but not parental GL-261 [88], suggesting that the immunizations specifically target the *IDH1*-mutant glioma cells. Indeed, the therapeutic responses are accompanied by increased CD8+ T cell populations and higher production of interferon (IFN)-gamma [88]. Despite observed discordance between CD4+ and CD8+ T cell activation, both preclinical models significantly increase the antitumor action in vivo. Based on the preclinical evidence, a phase 1 trial of IDH1 peptide vaccine in *IDH1*-mutant and 1p19q-intact grade III-IV astrocytomas has been completed [89] (ClinicalTrials.gov NCT02454634). In addition, two clinical trials of IDH1 peptide vaccine and IDH1 R132H dendritic cell vaccine have been launched in Duke University (ClinicalTrials.gov NCT02193347) and Beijing Tiantan and Hebei Yanda hospital (ClinicalTrials.gov NCT02771301), respectively (Table 1).

### 3.3. DNA Methylation Inhibitors

DNA hypermethylation acts as a memory signal for altered gene expression, and thus DNA-demethylating drugs are required for long-term epigenetic reprogramming [90]. In particular, two prototypal DNA-demethylating agents, decitabine (DAC, trade name Dacogen, Eisai) and azacitidine (AZA, trade name Vidaza, Celgene), have been FDA-approved for treating patients with myelodysplastic syndrome (MDS). Both drugs are under active investigation in the treatment of solid tumors, including gliomas. These drugs are cytidine analogs that incorporate into the DNA in the case of both agents, and RNA in the case of azacitidine, and form an irreversible covalent bond with DNMTs, which triggers ubiquitin-dependent degradation of the enzymes [91,92]. Consequently, subsequent round of DNA replication induces significant demethylation in daughter cells, leading to reactivated expression of epigenetically repressed genes [93]. Numerous studies have shown that these DNMT inhibitors (DNMTis) allow cancer cells to reactivate the aberrantly silenced tumor suppressor genes by promoter demethylation [24,94]. In addition to promoter regions, the DNA methylation in intragenic regions is correlated with their transcriptional activity [95,96]. In line with this finding, AZA induces gene body DNA demethylation, resulting in reduced expression of genes regulated by MYC [97]. 

Of particular importance, DAC shows a U-shaped dose response curve of hypomethylation in a number of cancer types [98]. The hypomethylation effect is possibly mitigated by the fact that excessive doses of DAC induce DNA synthesis inhibition, cytotoxicity, and cell-cycle arrest [98,99]. In breast cancers and leukemia, low-dose DAC and AZA can exhibit a persistent antitumor response without causing early cytotoxicity and DNA damage [100]. Furthermore, the patterns of DAC or AZA-mediated clinical response are quite different from those of conventional chemotherapy [101]. For example, DAC or AZA induces relatively slow responses compared to cytotoxic agents, suggesting that these broad reprogrammers indirectly affect the clinical responses following epigenetic reprogramming beyond direct cytotoxicity. 

A robust and durable activity of DNMTi on tumor growth inhibition described by our group is that DAC-treated glioma cells harboring *IDH1* mutation and 1p19q co-deletion remain viable, but without evidence of tumor growth onset in mice for 55 days [83]. A similar, but independent study reported that long-term AZA treatment shows significant tumor regression in a mouse xenograft of *IDH1*-mutant astrocytoma model [102]. Overall, regardless of the presence of 1p19q codel, both studies demonstrated that DAC and AZA induce durable therapeutic effects in patient-derived *IDH1*-mutant gliomas without any signs of tumor re-growth despite termination of administration [83,102]. Based on the promising evidence seen in two preclinical studies, a phase 2 study of *IDH1/2*-mutant gliomas is being conducted to evaluate the efficacy of AZA on progression-free survival at 6 months (PFS-6) (ClinicalTrials.gov NCT03666559).

Given that DAC and AZA have short half-lives, the drug delivery, particularly in solid tumors, including gliomas, is one of the acknowledged barriers for effective therapy of DNA demethylating agents. Mechanistically, once in the cell, DAC and AZA are rapidly deaminated by the ubiquitously expressed cytidine deaminase (CDA), leading to a drastic reduction in their activities [103,104]. Because of their poor in vivo stability, much effort has been devoted to overcome this problem to achieve stable pharmacokinetics. Currently, a phase 1 study of *IDH1*-mutant gliomas is ongoing for determining the maximum tolerated dose (MTD) of ASTX727, which consists of DAC and E7727 (cedazuridine), a novel CDA inhibitor (ClinicalTrials.gov NCT03922555).

Because of inter-individual variability in clinical response [101], there are ongoing efforts to understand the novel mechanism of action induced by these drugs. Accumulating studies have revealed that DNMTis upregulate interferon signaling in cancer cells by double-stranded RNA (dsRNA) formation from re-expression of epigenetically silenced endogenous retroviruses (ERVs) [105,106]. In human breast cancers, SGI-110 (guadecitabine), a next-generation hypomethylating agent, induces the expression of major histocompatibility class-I (MHC-I) genes, leading to T cell-mediated responses and tumor regression in vivo [107]. Recent experimental evidence suggested that *IDH* mutation and 2-HG accumulation are closely associated with immunosuppressive tumor microenvironment in gliomas [29,30,108]. Zhang and Rao et al. reported that *IDH1*-mutant gliomas escape natural killer (NK) cell-mediated cytotoxicity by epigenetic silencing of NK group 2D (NKG2D) ligands [108]. Moreover, DAC treatment induces the expression of unique long 16 (UL-16)-binding proteins (ULBPs) as NKG2D ligands, and restores NK cells-mediated antitumor immune response [108]. In accordance with these findings, DAC induces the expression of several immune-related genes such as MHC-I, ICAM-1 [109], and cancer testis antigens (CTAs) [110], and sensitizes glioma cells to cytotoxic CD8 T cells (CTLs)-mediated antitumor immune response (Figure 2), providing the rationale for combined use of DNA hypomethylating agents with immunotherapy such as immune checkpoint inhibitors, as will be discussed below.

### 3.4. Combining DNMTi with Cytotoxic Drugs for Glioma

MGMT promoter methylation and its protein levels are known to determine the sensitivity of gliomas to TMZ [46,111]. In case of an unmethylated MGMT promoter, cytosine modification of the MGMT gene body positively correlates with the expression levels, and DAC induces hypomethylation along the MGMT gene body accompanied by its decreased gene expression [112]. Consistent with this finding, DAC treatment sensitizes GBM cells expressing MGMT to TMZ [112,113]. Recently, in *IDH1*-mutant gliomas, the therapeutic effect of AZA is further enhanced in combination with TMZ [114]. Yamashita et al. showed that the combination AZA and TMZ increases the DNA damage response corresponded with reduced cell viability. Despite AZA-mediated demethylation of *MGMT* promoter, AZA does not interfere with TMZ sensitivity in the combination setting [114]. In particular, combining AZA and TMZ significantly reduces tumor growth and extends the survival of mice in subcutaneous and intracranial xenografts of *IDH1*-mutant gliomas, respectively [114]. 

It has been revealed that the expression and activity of DNMTs is differentially regulated during cell cycle transition [115]. In addition to this, DAC and AZA are S phase-specific drugs. Determining the therapeutic efficacy of combining DNMTi with other cancer therapies such as standard cytotoxic drugs is an area of investigation.

### 3.5. Combination of Epigenetic Drugs and Immunotherapy

*IDH*-mutant gliomas exhibit a profound immunosuppressive microenvironment [116]. The oncometabolite 2-HG can be detected in the cerebrospinal fluid of patients harboring *IDH*-mutant tumors [117], suggesting that extracellular 2-HG may impact the tumor microenvironment, including non-malignant cell types such as T cells, microglia and macrophages. Indeed, to date, several mechanisms by which tumor cell-derived 2-HG promotes immune evasion have been identified [30,118]. 

An increasing number of studies demonstrated that the suppression of immune response in tumor microenvironment is generally achieved by programmed death 1 (PD1) or cytotoxic T lymphocyte-associated protein 4 (CTLA4) on T cells [119]. Interestingly, *IDH*-mutant gliomas display lower tumor infiltrating lymphocytes (TILs) and reduced *PD-L1* expression, possibly because of PD-L1 promoter methylation [120]. Consistent with this, introduction of *IDH1* mutation or exogenous 2-HG in syngeneic mouse glioma models reduces the levels of CTL-attracting and IFN-gamma-inducible chemokines, including CXCL10 [121]. Remarkably, IDH-C35, a mutant IDH1-specific inhibitor, in combination with a peptide-based vaccine immunotherapy significantly improves the survival of mice bearing GL261 R132H gliomas compared to vaccine alone, providing evidence that epigenetic agents may sensitize patients to concurrent or subsequent immune therapy. Most recently, a phase 1 trial in gliomas has been launched to determine the safety and tolerability of IDH1 R132H peptide vaccine, PD-L1 checkpoint inhibitor (Avelumab) or their combination (ClinicalTrials.gov NCT03893903). 

As discussed above, a growing body of evidence has shown that DNMTis can augment immune attraction properties of cancer cells [122,123]. Notably, a 317 gene expression signature named as AZA-induced immune genes (AIMs) has been identified by using 63 cancer cell lines following AZA treatment. In gliomas, AZA upregulates CTAs, including melanoma-associated antigen D4 (*MAGED4*) [124]. Similar results were observed that AZA triggers the induction of NY-ESO-1, highly immunogenic CTAs, in orthotopic glioma xenografts [125]. An exciting recent study showed that robust upregulation of NY-ESO-1 by DAC, combined with NY-ESO-1-specific adoptive T cell-based immunotherapy, displays encouraging antitumor activity, and confers substantial survival benefit to mice bearing intracranial glioma xenografts [126]. Therefore, a combination of epigenetic drugs and immunotherapy holds a great promise for potential therapeutic strategies capable of eradicating glioma cells (Figure 2).

## 4. Conclusions

In view of high degree of inter- and intratumoral heterogeneity in gliomas, more precise therapeutic targeting of molecularly defined subgroups or individuals will be required. In line with this, epigenetic analysis such as DNA methylation sequencing needs to be implemented as a powerful tool for characterizing the heterogeneity and for informing clinical decisions. Recent work from Capper et al. exquisitely shows the power of methylation-based classification to improve the diagnostic accuracy of CNS tumor entities. Importantly, this approach also has the potential to reveal previously unrecognized brain tumor entities [127]. To maximize this potential, ongoing research efforts continue to advance our understanding of the pathological epigenetic events in glioma development. In particular, altered epigenetic mechanism is a major consequence of *IDH* mutations and 2-HG accumulation, which can be a vulnerable target for epigenetic therapy of *IDH*-mutant gliomas. The efficacy of epigenetic drugs on tumor reduction can feasibly be monitored in patients using 2-HG as a surrogate marker for tumor progression. Indeed, several studies have shown that 2-HG levels can be noninvasively measured by magnetic resonance spectroscopy in patients [128,129,130]. However, more studies are needed to better understand the mechanisms underlying the actions of epigenetic drugs on gliomas. For example, it remains to be seen whether DNMTi-driven global DNA hypomethylation induces non-specific effects such as genetic instability, particularly at high doses. Notably, these epigenetic modulators can be taken up by non-malignant cells, including immune cells. Therefore, the effects of epigenetic drugs on the glioma tumor microenvironment need to be elucidated. Additionally, it will be important to clarify whether *IDH* mutations contribute to gliomagenesis through a “hit and run” mechanism. 

Here, we highlight the promising antitumor activity of epigenetic drugs alone or in combination, although the impact of treatment schedule in the combinatorial strategies, sequential or concurrent, is not fully understood. Given that synergistic effects can be obtained from the combination settings with immunotherapy and different epigenetic therapies, its proper integration into a combined strategy may deliver the long-sought breakthrough in glioma therapy.

## Figures and Tables

**Table 1:** Ongoing epigenetic trials in *IDH*-mutant gliomas.

**IDH inhibitor**						
**Drug**	**Clinical Trials** **gov identifier**	**Phase**	**Cancer Type**	Enrollment	**Primary Endpoint(s)**	**Sponsor(s)**
AG-120 (Ivosidenib)	NCT02073994	1	*IDH1* mutant malignancies	170	Safety and tolerability, MTD and/or RP2D	Agios
AG-881	NCT02481154	1	*IDH1* and/or *IDH2* mutant malignancies	150	Safety and tolerability, MTD and/or RP2D	Agios
AG-120 AG-881	NCT03343197	1	*IDH1* mutant gliomas	45	2-HG concentration	Agios
DS-1001b	NCT03030066	1	*IDH1* mutant gliomas	60	DLTs	Daiichi Sankyo Co.
IDH305	NCT02381886	1	*IDH1* mutant malignancies	166	DLTs	Novartis
FT-2102	NCT03684811	1b/2	*IDH1* mutant malignancies	200	DLTs, RP2D (phase 1) ORR of FT-2102 single agent or in combination with azacytidine (phase 2)	Forma
BAY1436032	NCT02746081	1	*IDH1* mutant malignancies	81	Safety and tolerability, MTD and/or RP2D	Bayer
**IDH1 peptide vaccine**						
**Vaccine**	**Clinical Trials** **gov identifier**	**Phase**	**Cancer Type**	**Enrollment**	**Primary Endpoint(s)**	**Sponsor (s)**
PEPIDH1M	NCT02193347	1	*IDH1* mutant gliomas	24	Safety and tolerability	Gary Archer
IDH1R132H- DC	NCT02771301	NA	*IDH1* mutant gliomas	30	Safety and efficacy	Hebei Yanda Hospital
IDH1R132H peptide vaccine	NCT03893903	1	Malignant glioma	60	Safety and tolerability	CGRC
**DNMT inhibitor**						
**Drug**	**Clinical Trials** **gov identifier**	**Phase**	**Cancer Type**	**Enrollment**	**Primary Endpoint(s)**	**Sponsor (s)**
Azacitidine (Vidaza)	NCT03666559	2	*IDH* mutant gliomas	63	Progression-Free Survival at 6 months (PFS-6)	AP-HP
ASTX727	NCT03922555	1	*IDH* mutant gliomas	18	MTD	MGH

Abbreviation: AP-HP, Assistance Publique-Hôpitaux de Paris; CGRC, German Cancer Research Center; DC, dendritic cells; DLTs, dose limiting toxicities; MGH, Massachusetts General Hospital; MTD, maximum tolerated dose; NA, not applicable; ORR, objective response rate; RP2D, recommended phase II dose.
